# Bacterial and Fungal Pathogens: New Weapons to Fight Them

**DOI:** 10.3390/antibiotics13050384

**Published:** 2024-04-24

**Authors:** Charlotte A. Huber

**Affiliations:** Centre for Clinical Research, The University of Queensland, Herston, Brisbane, QLD 4029, Australia; ca.prestonhuber@gmail.com

In high-income countries, degenerative diseases are the primary cause of death. In the past, however, most people worldwide were killed by infections and did not live long enough to die from degenerative diseases. Life expectancy was much lower, and in many cases, infant mortality exceeded ten percent.

One highly virulent pathogen was *Yersinia pestis*, the causative agent of the plague. It is considered to have killed between one- and two-thirds of Europe’s population in one of its pandemics (1347–1352). Centuries later, but still in the pre-antibiotic era, the plague was held at bay by human intervention. Nowadays, the plague is treatable with antibiotics and is no longer such a menace [1]. 

The introduction of antibiotics has revolutionised modern medicine. In addition to the treatment of infectious diseases, antibiotics are used for prevention during medical procedures, such as cancer treatment and surgery. However, bacteria have been around for billions of years and have developed methods to evade compounds that are toxic to them. Extensive use of antibiotics has resulted in selection pressure in favour of resistant strains, causing the prevalence of antibiotic resistance to rise steadily. As a result, we run the risk of approaching a post-antibiotic era, and some pandrug-resistant bacterial strains are already untreatable. 

Decades ago, the emergence of resistance was met with the introduction of new antibiotics into clinical practice. More recently, a lack of success and rising costs have resulted in large pharmaceutical companies discontinuing antibiotic discovery and development. As a result, few candidates are in the clinical trial pipeline while resistance continues to evolve and disseminate. Deaths associated with antibiotic resistance have exceeded one million, and, unless the situation improves, millions more are expected to die from previously easily treatable infections. New weapons to combat bacterial infections are imperative [2].

This Special Issue sought contributions on molecular methods in antibiotic discovery. Eleven papers were published, describing novel compounds, formulations, or repurposed drugs (https://www.mdpi.com/journal/antibiotics/special_issues/Molecular_Methods; accessed on 30 March 2024).

Most papers describe novel compounds, including homopolymers (contribution 1), cyclic imides (contribution 2), antimicrobial peptides (contribution 3), nucleic acids (contribution 4), and derivatives of quinone (contribution 5), benzamidine (contribution 6), thiazole (contribution 7), and pyrazole (contribution 8). Two papers describe novel galenic formulations of titanate (contribution 9) or silver nanoparticles (contribution 10), respectively. One contribution describes repurposed drugs (contribution 11).

Fungi, being eukaryotes, are distinctly different from bacteria. However, drug resistance has also become a concern for fungal infections, particularly in the immunocompromised. Resistance to antimicrobials is prevalent among several microbial kingdoms. Although systemic treatment of fungal infections has relied on only four classes of antifungals, fungi have been neglected when aiming to address the threat of antimicrobial resistance [3]. Furthermore, fungi are able to build interkingdom biofilms with bacteria, potentially worsening the outcome. While most of the contributions to this Special Issue describe compounds with antibacterial activity, two papers describe antimicrobial candidates that have both antibacterial and antifungal properties (contributions 7 and 9), and one paper describes compounds with antifungal activity (contribution 3).

Biofilms protect microorganisms from external influences, such as host immune responses and pharmaceuticals. This further exacerbates the problem of antimicrobial resistance. Three papers contributing to this Special Issue describe compounds (contributions 3 and 8) or formulations (contribution 10) with antibiofilm activity.

In vitro toxicity testing using cell lines was performed in many cases. Although describing a novel antibacterial mechanism of action, one contribution to this Special Issue describes repurposed drugs. Having undergone extensive safety testing, including in humans, repurposed drugs can undergo clinical trials relatively quickly and inexpensively (contribution 11).

Antimicrobials in preclinical research are innovative and diverse. However, less than 50 antibiotic candidates were in the clinical trial pipeline in December 2020. At the same time, the number of candidates for cancer treatment was estimated to be in excess of 1300. Not only did large pharmaceutical companies discontinue the development of new antibiotics, but smaller companies that took over suffered great financial loss or even insolvency. Paradoxically, this happened upon successful introduction of their product into clinical practice. New antibiotics are often inexpensive, and being used as last-line options, they have low sales volumes. A satisfactory return on investment cannot be guaranteed, even if a product successfully reaches the market. Therefore, antibiotic candidates are abandoned because of a lack of funding in preclinical and early clinical research stages (referred to as the “valleys of death”). What complicates the issue even further is that many experts in antibiotic development have retired, and there is little financial incentive for young scientists to enter the field. 

Meanwhile, the development of novel antifungals faces similar issues. The clinical trials necessary for market approval are time-consuming and may cost hundreds of millions of US dollars. In the case of antifungals, too, it is a challenge to reach a market big enough to make a development project financially viable [3].

It has become clear that classic financing models give little incentive for the pharmaceutical industry to develop new antimicrobials. However, some funding strategies have been suggested, such as grants for basic antimicrobial drug discovery and preclinical research, and market entry rewards. 

Finding new weapons to fight bacterial and fungal pathogens is of great urgency and importance to the public. However, free-market principles may not get us there, and we may instead depend on foundations and well-invested taxpayers’ money [2,3,4].
